# Racial differences in prostate cancer: does timing of puberty play a role?

**DOI:** 10.1038/s41416-020-0897-4

**Published:** 2020-05-22

**Authors:** Jinhee Hur, Edward Giovannucci

**Affiliations:** 1 Department of Nutrition, Harvard T.H. Chan School of Public Health, Boston, MA USA; 2 Department of Epidemiology, Harvard T.H. Chan School of Public Health, Boston, MA USA; 3 Channing Division of Network Medicine, Brigham and Women’s Hospital and Harvard Medical School, Boston, MA USA; 4 Department of Medicine, Harvard Medical School, Boston, MA USA

**Keywords:** Risk factors, Prostate cancer

## Abstract

The burden of prostate cancer has a remarkably disproportionate distribution across racial groups. For example, in the USA, African Americans are twice as likely as individuals of European ancestry to develop or die from prostate cancer, and have a more aggressive disease nature at diagnosis. In contrast, Asian American men have the lowest incidence and mortality rates of prostate cancer. That considerable racial disparities exist even in the subclinical stage of prostate cancer among young men in their 20–30s suggests that patterns of prostate carcinogenesis start to diverge even earlier, perhaps during puberty, when the prostate matures at its most rapid rate. Mendelian randomisation studies have provided strong population-based evidence supporting the hypothesis that earlier onset of puberty increases the risk of prostate cancer—particularly of high grade—and prostate cancer-specific mortality later in life, observations which correspond to the epidemiology of the disease in African Americans. Notably, African American boys initiate genital development ~1 year earlier and thus go through longer periods of pubertal maturation compared with European American boys. In this perspective, bringing together existing evidence, we point to puberty as a potential critical window of increased susceptibility to prostate carcinogenesis that could account for the marked prevailing racial differences in the burden of prostate cancer.

## Background

A considerable racial disparity in the incidence and mortality rates of prostate cancer is a well-recognised public health concern. In the USA, for example, African Americans have the highest incidence and mortality rates, approximately twice as high as those of European American men and even three or four times higher than those of Asian Americans (Table 1).^1^ Moreover, prostate cancers diagnosed in African Americans tend to be of a more aggressive nature than those of European Americans; and African American men are more likely to have advanced or metastatic disease at diagnosis.^2^ Similar high incidence and mortality rates are also seen in men of African descent in areas other than the USA, such as Jamaica.^3,4^ Extensive speculation exists regarding potential reasons for this disparity, including genetic factors, socioeconomic status and environmental factors, among others. Differences in the availability of screening programmes and access to healthcare might also contribute in part to the discrepancy, but cannot account for the 2-fold increase in incidence and mortality rates.^5^ Relatively few risk factors are established for prostate cancer and, to date, no obvious risk factor appears to account for these racial differences.

**Table 1:** Racial difference in incidence and mortality rates for prostate cancer in the USA, 2012–2017^a,b^.

	Incidence, 2012–2016	Mortality, 2013–2017
All races combined	104.1	19.1
African American	173.0	38.7
European American	97.1	18.0
Asian/Pacific Islander	52.9	8.6
Hispanic	86.8	15.7
American Indian/Alaska Native^c^	68.0	18.7

^a^Derived from Siegel et al.^1^

^b^Rates are per 100,000 population and age-adjusted to the 2000 US standard population. Racial categories other than African American and European American are not mutually exclusive of Hispanic origin.

^c^Data based on Purchased/Referred Care Delivery Area (PRCDA) counties and exclude data from Kansas and Minnesota.

A distinctive feature of the epidemiology of these racial differences in incidence and mortality rates is that whatever accounts for them tends to occur earlier in life, such that African American men tend to have an earlier onset of prostate cancer^6^ as well as being at higher risk of the disease. Even in the case of undiagnosed prostate cancer detected by histology at autopsy, this disparity is consistently observed across ages and is particularly strong at younger ages (i.e. 20–39 years),^7^ indicating an earlier onset of prostate carcinogenesis in African Americans. Amidst the dearth of compelling modifiable risk factors for prostate cancer, growing evidence points to the importance of aetiological factors early in life, when the immature and growing prostate could be more susceptible to carcinogenic exposures.^8,9^ Putting together what we know so far, we hypothesise that the earlier onset of puberty in males could be a risk factor for prostate carcinogenesis and potentially account for the observed racial disparities in the disease burden.

## The onset of puberty, and factors that influence it

Puberty is an important milestone, representing the transition from childhood to adulthood and the achievement of reproductive capability. Puberty commences with reactivation of the hypothalamic–pituitary–gonadal axis, which has been largely dormant since shortly after birth. Although the underlying mechanisms that regulate pubertal onset remain to be completely delineated—particularly in males—there seems to be substantial overlap in the underlying biology between the timing of male and female pubertal development, including common genetic determinants.^10,11^

Boys progress through puberty in the sequence of genital development, pubic hair growth and peak height velocity.^12^ Facial hair growth and voice deepening usually take place around the time when pubertal growth spurt begins.^13^ Testicular enlargement and changes in the colour and texture of the scrotal skin, indicated by Tanner stage 2 of genital development, have been considered to be the first sign of pubertal onset in boys, which typically starts between the ages of 10 and 14 (Table 2).^14^ Pubertal maturation is more difficult to assess in boys than in girls, primarily due to the lack of a salient and thus memorable milestone such as menarche. Visual evaluation of testicular volume is difficult, but can be assessed in clinical settings using an orchidometer; however, the relatively intrusive and subjective nature of assessment can still curtail patient/study participant-friendly and practical use to ascertain and generate data on male pubertal development.

**Table 2:** Tanner stages for genital development and pubic hair growth in boys^a^.

Tanner stage	Genital development		Pubic hair growth	
	Definition	Age range	Definition	Age range
1	Prepubertal stage; testes, scrotum and penis are of about the same size and proportion as in early childhood.		Prepubertal stage; no pubic hair, the fine hair is not further developed than that over the abdomen.	
2	The scrotum and testes have enlarged and there is a change in the texture and reddening of the scrotal skin.	9.5–13.8	Sparse growth of long, slightly pigmented, downy hair, straight or slightly curled, appearing mostly at the base of the penis.	11.3–15.6
3	Growth of the penis has occurred, at first mainly in length but with some increase in breadth. There has been further growth of testes and scrotum.	10.8–14.9	Considerably darker, coarser and more curled hair starts to spread sparsely over the pubic area.	11.8–16.0
4	Penis further enlarged in length and breadth with development of glans (wider area at the end of penis). Testes and scrotum further enlarged. There is also further darkening of the scrotal skin.	11.7–15.8	Hair is now more likely in adult type, but the area covered by the hair is still considerably smaller than in most adults. There is no spread to the inner thighs.	12.2–16.5
5	Penis and scrotum are now in adult size and shape. No further enlargement takes place after stage 5 is reached.	12.7–17.1	The quantity and type of the hair is now similar to that of adults. It is distributed as an inverse triangle pattern and spreads to the inner thighs, but not elsewhere above the base of the inverse triangle.	13.0–17.3

^a^Derived from Marshall and Tanner.^14^

Pubertal traits are heritable, and several genes controlling the reproductive axis have been identified.^15^ Large-scale genomic studies, mostly carried out in females, identified a substantial number of new loci associated with the timing of puberty (i.e. menarche).^16^ However, these loci could account for only ~7% of the variance in age at menarche, equivalent to ~25% of heritability, which, in turn, might point to the importance of environmental cues interacting with—or possibly overriding—genetic predisposition in determining the onset of puberty.

Regarding environmental factors that influence the timing of puberty, nutrition, socioeconomic status, stress and exposure to endocrine-disrupting chemicals have been identified as potential candidates.^17^ Of nutritional factors, evidence has consistently indicated the role of childhood adiposity and protein intake in the onset of puberty.^18^ Compared with the well-established link in females between body fat and age at menarche, epidemiological findings have been mixed for male pubertal onset.^19^ Intake of total and animal protein at age of 5–6 years was inversely associated with, whereas that of vegetable protein at age of 3–4 years was positively associated with several pubertal milestones such as ages at voice break in boys, menarche in girls and pubertal growth spurt and peak height velocity in both sexes.^20^ Within the US population, a positive socioeconomic gradient in age at menarche (that is, a lower socioeconomic status associated with earlier age at menarche) was observed among European American but not in African American girls, and could be possibly attributable to the increasing prevalence of childhood obesity (and, consequently, body fat) among European American girls of low-income families.^21^

As endocrine-disrupting chemicals have oestrogenic and/or antiandrogenic effects, it has been postulated that exposure to these compounds might disrupt hormonal balance and thus alter timing of puberty; however, in part due to the huge variability in types, dose and window of exposure to a wide array of chemicals, findings have been inconsistent and data are much more limited in boys.^22,23^ Although these factors may play a role, age of earlier puberty has been lowering dramatically and uniformly in diverse populations beginning from at least 1830,^24,25^ indicating that nutritional status and related factors have the dominant role.

Psychological anxiety due to absence of a biological father was associated with an earlier timing of puberty in US boys and girls, independent of socioeconomic status.^26^ A prospective study of US girls also supported the link between absence of a resident biological father and earlier breast development among girls from high-income families and pubic hair growth only among African American girls from high-income families, and this association remained significant when body mass index was taken into account.^27^ Taken together, this suggests that intervening exposure to these environmental factors might moderate pubertal timing beyond well-recognised factors related to energy homoeostasis, such as childhood overweight and obesity.

## Puberty as a potential window of increased susceptibility for prostate carcinogenesis

Puberty is the period of time over the course of a man’s life when the prostate matures most rapidly, more than doubling in volume within 6 months to 1 year.^28^ During pubertal progression, the immature epithelium of the prostate gland undergoes extensive cell proliferation and differentiation, mainly into basal, luminal secretory and neuroendocrine cells.^29,30^ Of these cells, the luminal secretory cells share identical immunohistochemical characteristics with prostate carcinoma cells.^29^ Nearly all (>95–99%) cases of prostate cancer are known to be derived from luminal cells,^30^ suggesting that puberty, when these cells start to appear, could constitute a particular window of increased susceptibility for prostate carcinogenesis.

Data estimating the doubling times of prostate cancer cells have provided mathematical evidence supporting the hypothesis that the proliferation of prostate tumour cells can start as early as 14 years of age^31^—around the time of puberty. Although clinical forms of prostate cancer manifest later in adulthood, histological prostatic intraepithelial neoplasia and prostate cancer can be detected at autopsy as early as the third decade of life,^7^ consistent with the causative factors occurring very early in life, perhaps at puberty. Thus, various lines of data based on the timing of key pathological events converge to indicate that puberty might be a critical time for the initiation of prostate cancers.

Why puberty is a particularly significant period of susceptibility for prostate cancer, however, is not entirely clear from a mechanistic basis. Notably, the onset of puberty initiates dramatic changes in the hormonal milieu, with circulating levels of androgens and insulin-like growth factor-1 (IGF-1) being elevated to regulate normal somatic growth and sexual maturation. A longitudinal study demonstrated a strong correlation between age at peak velocity for the rise in serum IGF-1 levels and pubertal characteristics in boys such as age at peak height velocity (*r* = 0.92) and Tanner stages for genital development and pubic hair growth (*r* = 0.77).^32^ In addition, a greater, prepubertal IGF-1 level at age of 10 years was associated with an earlier onset of puberty.^32^ Based on annual evaluations of endocrine profile over the course of the pubertal transition, compared with their European American peers, African American boys had higher levels of IGF-1 during the prepubertal period and lower concentrations of its binding protein (IGFBP)-1 and IGFBP-3 throughout puberty, indicating higher levels of bioavailable IGF-1 in African American boys during puberty.^33^

In turn, accumulating evidence consistently points to a cancer-enhancing effect of IGF-1, a major growth-regulating hormone and an inhibitor of apoptosis, on the prostate.^34^ A large pooled meta-analysis of individual participant data from 17 prospective and two cross-sectional studies, including up to 10,554 prostate cancer cases and 13,618 controls, demonstrated a clear association between circulating IGF-1 levels and the risk of prostate cancer, especially for individuals diagnosed before the age of 60 years.^35^ Earlier onset of puberty was associated with a greater serum level of IGF-1 in men in their 60s, suggesting the biological programming of the IGF-1 system might plausibly take place during puberty, although the precise mechanism for this remains to be elucidated.^36^ In the middle-aged, US male population, individuals of African ancestry had the lowest IGFBP-3 level, followed by those of European and Asian ancestries, which coincide with the well-known racial disparity in the incidence and mortality rates of prostate cancer.^37^ Notably, high IGF-1 levels and low IGFBP-3 levels, less consistently for the latter, have been associated with an increased risk of prostate cancer, particularly of a more advanced stage.^38,39^ Identification of genetic variants in IGF-1 associated with prostate cancer risk in multi-ethnic populations also lends support to the potential, important role of IGF-1 in prostate carcinogenesis.^40^

## Epidemiological evidence that links onset of puberty and risk of prostate cancer

From a biological perspective, as has just been described, puberty provides a potential window of susceptibility for prostate cancer. An obvious question, therefore, is whether there is an association between the timing of male puberty and the risk of developing prostate cancer later in life? A well-accepted, analogous example in females is the association between earlier age at menarche and increased risk of breast cancer.^41–43^ However, in part due to the lack of such a landmark pubertal milestone in boys, there is limited evidence regarding male pubertal characteristics that might be associated with health consequences later in life, particularly prostate cancer. Minimal research, mostly from case-control studies, has used other characteristics (e.g. ages at first sexual intercourse, shaving and voice break) that have been considered a proxy for the onset of male pubertal maturation to implicate a protective effect of later puberty on the risk of prostate cancer, but the findings have been largely inconsistent and possibly prone to recall bias.^44–48^

The results of a study carried out in 2016^49^ provide strong support for an association between the timing of puberty and the risk of developing prostate cancer later in life. Specifically, the evidence was derived from a Mendelian randomisation study using a genetic score combining 13 single nucleotide polymorphisms associated with male Tanner stages—with a higher score indicating later onset of puberty—in relation to prostate cancer risk.^49^ The association of this score with prostate cancer risk, stage and grade was examined in the UK-based ProtecT (Prostate testing for cancer and Treatment) case-control study (*n* = 2927), and the PRACTICAL (PRostate cancer AssoCiation group To Investigate Cancer-Associated aLterations in the genome) consortium (*n* = 43,737) was used as a replication sample. In ProtecT, the puberty genetic score was inversely associated with prostate cancer grade (odds ratio [OR] of high- versus low-grade cancer, per tertile of the score: 0.76; 95% confidence intervals [CI]: 0.64–0.89). In an instrumental variable estimation of the causal OR, boys with later pubertal maturation (equivalent to a difference in one Tanner stage between peers of the same age) had 77% (95% CI: 43–91%) decreased odds of high Gleason grade prostate cancer. In the PRACTICAL consortium, a 38% (95% CI: 22–51%) lower risk of 15-year prostate cancer-specific mortality was observed per unit difference in Tanner stage at the same age. Assuming an equivalent per-year effect between genetic variants associated with age at menarche in girls and age at voice break in boys,^11^ another Mendelian randomisation analysis that used the 1000 Genomes Project-imputed genotype data of 329,345 women also supported a protective effect of a later pubertal onset on the prostate cancer risk (OR: 0.92; 95% CI: 0.88–0.98), independent of body mass index.^16^

Mendelian randomisation exploits genetic variants as a proxy to an exposure or a risk factor of interest. Presumably, as genetic variants are less prone to biases such as confounding and reverse causation, this approach helps to derive a strong, causal inference with an outcome of interest.^50^ The findings from both Mendelian randomisation analyses showed no indication of directional pleiotropy, suggesting that the genetic variants were unlikely to influence the outcome (i.e. prostate cancer) via pathways other than the exposure (i.e. puberty) and thus the overall causal estimate was unlikely to be biased. The large size of these studies and robust findings confirm that the timing of pubertal onset is a strong risk factor for prostate cancer.

## Racial differences in the timing of puberty among US boys

If an earlier onset of puberty increases the risk of prostate cancer, does the timing of puberty differ by racial status such that an earlier onset of puberty in African Americans could account for the higher incidence and mortality rates observed? In the USA, racial differences in the timing of puberty, as assessed by Tanner stages, have indeed been observed among boys, as in girls.^51–53^ In one large study, practitioners collected data on Tanner stage and testicular volume among 4131 boys seen for well-child care in 144 paediatric offices across the USA.^51^ Compared with their European American peers, African American boys had an ~1-year earlier onset of genital development and pubic hair growth, as assessed by Tanner stage.^51^ The median age, however, for achieving the fully matured stage was comparable between the two sets, indicating that African American boys undergo pubertal maturation with an earlier onset but over a longer duration; such results were also consistently observed in a multicentre, longitudinal study of annual pubertal examinations.^52^

Whether Asian American boys who have the lowest prostate cancer risk mature later than their African American peers remains to be investigated; however, this pattern was observed for the pubertal timing of US girls. In a multi-ethnic prospective cohort study, African American girls had the earliest onset of breast development—the first sign of pubertal maturation in girls—followed by their peers of Hispanic, European and Asian ancestries,^54^ as well as a largely similar pattern for menarche.^55^ Considering the substantial overlap in the underlying biology between the timing of male and female pubertal development,^10,11^ this might be the likely pattern that exists for the racial differences in the timing of puberty between African and Asian American boys.

As noted above, the onset of puberty initiates dramatic changes in the circulating levels of androgens and IGF-1, which might contribute to prostate carcinogenesis.^34^ Of potential relevance, compared with their European American peers, African American boys had higher levels of IGF-1 during the prepubertal period and lower concentrations of its binding proteins throughout puberty, suggesting potential racial disparities in the IGF-1 system during this critical time period of prostate development.^33^ Thus, evidence demonstrates that African Americans have a notably earlier onset and longer duration of puberty along with hormonal changes that might be relevant for an increased risk of prostate cancer later in life.

## Earlier onset of puberty as an explanation for the higher rates of aggressive prostate cancer in African Americans

**Fig. 1:**
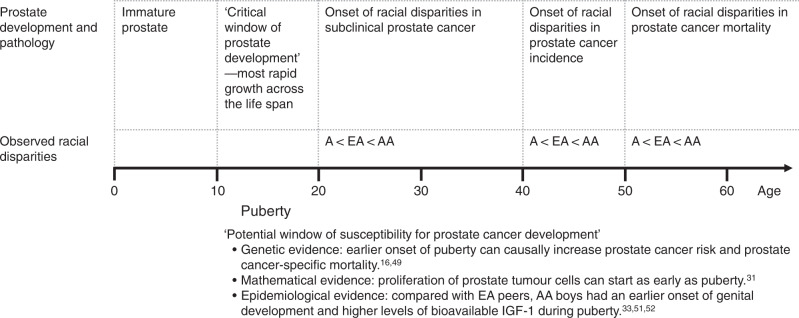
Puberty as a potential window of susceptibility for prostate cancer development. Abbreviations: A Asian; AA African American; EA European American, IGF-1 insulin-like growth factor-1.

Bringing together all the relevant evidence, we hypothesise that an earlier onset of puberty could increase the risk of developing aggressive forms of prostate cancer later in life, for which there are marked racial differences in the USA (Fig. 1). The results from the Mendelian randomisation studies provide strong genetic evidence that an earlier onset of puberty causally increases the risk of prostate cancer—in particular, aggressive forms—and prostate cancer-specific mortality.^16,49^ Moreover, the magnitude of the association, as indicated by the genetic variants, is strong. The second key piece of evidence is that African American boys show a substantially earlier onset of genital development than European American peers.^51,52^ If we assume a causal relationship between the actual onset of puberty and the risk of developing aggressive prostate cancer, we would anticipate higher rates of aggressive and fatal prostate cancer in African Americans. Furthermore, these racial differences would be pronounced, occurring at the earliest ages when prostate cancer becomes clinically apparent, such as by the fifth or sixth decades of life. These are precisely the patterns we observe for racial disparities in the epidemiology of prostate cancer in the USA. In conjunction with the earlier onset and longer duration of pubertal maturation observed in African Americans, exposure to high levels of IGF-1 and low levels of IGFBP-3, which have been speculated to facilitate tumour invasion and metastases,^39,56^ during a critical window of prostate development could increase susceptibility to prostatic neoplasia, particularly of a more aggressive nature, leaving lasting, long-term effects on the prostate, and thus possibly account for the well-known racial differences in the burden of prostate cancer.

## Recommendations for future research and public health implications

Puberty is a remarkable life event of public health importance, yet it is largely overlooked in cancer research. Given the complicated and less understood nature of puberty—particularly in males—longitudinal monitoring with a standardised measure across nationally representative, multi-ethnic groups should help to characterise the key features of male puberty across different racial groups. Given the lack of a salient pubertal milestone in boys, derivation of a composite measure from multiple pubertal characteristics might help to better represent male puberty.^48^ Epidemiological or experimental studies elucidating the underlying biological mechanisms between the timing of puberty and the risk of developing prostate cancer are recommended. Considering the plausible role of IGF-1 in prostate carcinogenesis and a strong link between IGF-1 and diabetes,^57^ future studies could decipher the association between pubertal timing and risk of diabetes and prostate cancer. Identification of strong instrumental variables for the timing of puberty and implementation of a Mendelian randomisation study in the African American population would contribute to generating strong, causal evidence on the potential link between puberty and prostate cancer. A better understanding of the factors that influence the onset of puberty is also important, although altering the timing of pubertal onset will be difficult to achieve from a practical perspective. Nonetheless, if puberty is a critical time period during which the risk of prostate cancer starts to diverge, complementary approaches that modify factors that are associated with early puberty, such as diet and physical activity, could be taken. For example, research suggests that diet during adolescence might have a role in the prevention of breast cancer.^58,59^ Besides nutritional influences, other factors that might influence the plasticity of the timing of puberty, such as psychological anxiety or stress, could be other relevant targets for intervention.

We hypothesise that the earlier onset of puberty could increase the risk of prostate cancer, particularly of a more aggressive nature, later in life, and could therefore account for the well-known, substantial racial differences in the burden of prostate cancer in the USA. This hypothesis does not imply that other factors do not in some way contribute to the differences—for example, how access to care and treatment might affect clinical outcomes. Nonetheless, as summarised above, evidence suggests that some of the apparent racial disparities are likely to occur early in life, well before the influences of screening and treatments. More research is needed to corroborate our hypothesis, but the overall coherence of evidence helps to assure its plausibility. Ongoing research on the topic could help provide aetiologic clues to the racial disparity in morbidity and mortality of prostate cancer, and in the developmental origins of prostate cancer in general. It could further guide primary prevention and intervention strategies that convert the window of susceptibility during puberty into an opportunity for future investment in better health outcomes and well-being of the population.

## Data Availability

Not applicable.
